# Epitaxial growth of BaHfO_3_ buffer layer and its structure degeneration analysed by Raman spectrum

**DOI:** 10.1186/s40064-016-3563-9

**Published:** 2016-11-03

**Authors:** Jiahui Zheng, Feng Fan, Xiangfa Yan, Yuming Lu, Yu Liang, Chuanyi Bai, Zhiyong Liu, Yanqun Guo, Chuanbing Cai

**Affiliations:** 1 Shanghai Key Laboratory of High Temperature Superconductors, Department of Physics, Shanghai University, Shanghai, 200444 China; 2 Shanghai Creative Superconductor Technologies Co., Ltd, Fengxian District, Shanghai, 201401 China

**Keywords:** BaHfO_3_, Cap layer, Magnetron sputtering, Raman, Coated conductors

## Abstract

BaHfO_3_ (BHO) has been proposed as a new cap layer material for YBa_2_Cu_3_O_7−δ_ (YBCO) coated conductors. Highly c-axis oriented BHO cap layer has been deposited on ion-beam assisted deposition-MgO buffered Hastelloy tapes by direct-current-magnetron sputtering method. The epi-growth of BHO films combined with its properties is investigated in details. The degenerated cubic crystal structure of BHO film is confirmed by Raman spectrum analysis. XRD θ–2θ scan, φ-scan and ω-scan reveal an excellent c-axis alignment with good in-plane and out-of-plane textures for BHO cap layers. SEM and AFM investigations show BHO cap layer a dense and crack-free morphology. Subsequently pure c-axis orientation YBCO film was epitaxial grown on such BHO cap layer successfully, shown BaHfO_3_ a potential cap layer material for coated conductors.

## Background

Due to their high performance and the potential low cost of both raw materials and preparation techniques, coated conductors based on the high temperature superconductors REBa_2_Cu_3_O_7−δ_ (REBCO or RE123; RE = Y, Nd, Sm or other rare earth elements) are expected to be practical materials application at liquid nitrogen temperature. To overcome the weak link behavior of the RE123 grain boundaries and achieve high performance coated conductors, the basic requirement is to realize RE123 films texture on flexible substrates. Main techniques to achieve the required biaxially textured RE123 superconducting layer include the rolling assisted biaxially textured substrates (RABiTS) (Goyal et al. 1996; Norton et al. 1996; Hühne et al. 2007), ion beam assisted deposition (IBAD) (Iijima and Matsumoto 2000; Muroga et al. 2003) and inclined substrate deposition (ISD) (Ma et al. 2004). Compared with RABiTS technique which needs metal substrate a texture structure first, IBAD technology can form the textured films on non-textured polycrystalline metal substrates and is used in worldwide.

Typically, coated conductor architecture includes three layers: the metallic substrate, the buffer layers and the RE123 superconductor layers. The buffer layers on the metallic substrate have two main functions for coated conductors based on the IBAD technique: (1) to form the texture structure and transfer the texture to the epi-grown RE123 superconductor layers; (2) to act as a barrier and prevent Ni or O atoms diffusion during the RE123 processing at high temperature.

Different oxide materials, including LaMnO_3_ (Aytug et al. 2003), CeO_2_ (Bhuiyan and Paranthaman 2003), SrTiO_3_ (Sathyamurthy and Salama 2002) and TiN (Xiong et al. 2010), have been successfully used as buffer layers to fulfill these requirements. Although the mechanism is not clear, it was found that different cap layer properties including material, texture or surface morphology affect subsequently RE123 epi-growth and performance seriously (Wang et al. 2004). Development of new cap layer material is helpful to understand these influences.

Recently, BaHfO_3_ was found exist stably in RE123 films and was researched as a dope to improve the superconductivity properties of coated conductor films (Erbe et al. 2015). Moreover, no significant reaction between YBCO and BaHfO_3_ was detected up to YBCO melt temperature about 1060 °C in melt-texture growth technique (Zhang and Evetts 1994). In this case, BHO was thought to be an excellent cap layer material for coated conductors. In this paper, BaHfO_3_ was deposited on IBAD-MgO buffered Hastelloy substrate as cap layer via DC-magnetron sputtering technique. The biaxially textured BHO cap layer shows a dense, smooth, and crack-free morphology. Finally the YBCO films were epitaxially grown on the BHO cap layer via pulsed laser deposition (PLD) method, shown BHO a new potential buffer material.

## Experiments

Hasterlloy tapes were electro-polished to a surface roughness less than 2.0 nm and then used as metal substrate. None crystallized Al_2_O_3_ and Y_2_O_3_ were deposited by Ion-Beam technique at room temperature as barrier layer and seed layer, respectively. MgO template layers were then deposited by ion beam assisted deposition technique (IBAD) to form the biaxial texture.

BaHfO_3_ (BHO) layers were then epi-grown on such MgO buffered tapes in a reel-to-reel magnetron sputtering system to form BHO/IBAD-MgO/Y_2_O_3_/Al_2_O_3_/Hastelloy structure. Metal Barium (99.9%) and Hafnium (99.95%) spliced with area ratio about 1:1 were used as metal targets for BHO cap layers deposition. The BHO was deposited in Ar/O_2_ mixture atmosphere with a total background pressure of 1 Pa and an oxygen partial pressure of about 0.15 Pa. The thickness of BHO films are about 40–50 nm, which are measured with a surface profiler (DEKTAKXT, Bruker Corporation).

After that, typical 300 nm thick YBCO films were epi-grown by pulsed laser deposition (PLD) method to check up the performance of the BHO cap layers with a substrate temperature of 810 °C, a background oxygen pressure of 30 Pa and an oxygen loading step under 4 × 10^4^ Pa during cooling down. More details can be found elsewhere (Hühne et al. 2007; Ying et al. 2009).

Raman scattering experiments were performed in back-scattering geometry using a Spex 1403 double monochromator equipped with a RC31034 photomultiplier and photocounting system to character the fabricated BHO films and analysis their crystal structure. A Spectra-Physics 171 argon ion laser with a 488 nm laser line was served as the excitation source at an incident power of 10 mW on samples.

To evaluate the textures and the orientation of BHO and YBCO films, X-ray diffraction (XRD, Philips X’Pert PRO, Cu Kα, λ = 1.54185 Å) analysis including θ–2θ scan, φ-scan, and rocking curve were performed. The surface morphology and micro-structures of the BHO films were investigated by Scanning Electron Microscope (SEM, Apollo 300) and Atomic Force Microscopy (AFM, Nanofirst 3600A), respectively. The critical current density J_c_ of the YBCO coated conductor was examined by inductive measurement using a Cryoscan by Theva.

## Results and discussion

**Fig. 1:**
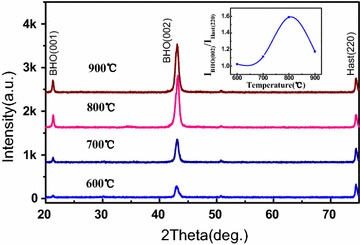
XRD θ–2θ pattern of BHO films deposited at different substrate temperature

Different substrate temperatures ranging from 700 to 900 °C were used to evaluate the epitaxial growth of BaHfO_3_ on IBAD-MgO buffered substrate. Figure 1 shows the typical θ–2θ XRD pattern of BaHfO_3_ cap layers. They demonstrate that no additional phases are formed except the desired BHO films. Only BHO (001) and BHO (002) diffraction peak were visible. The BHO films show (001) reflections only indicating a strong (001) texture for all substrate temperatures investigated. Additionally, the intensity ratio of *I*_BHO (002)_ and *I*_Hast (220)_ reflections is calculated to evaluate the growth of BHO films, shown in the inset. The ratio of *I*_BHO (002)_/*I*_Hast (220)_ increases with substrate temperature rise, which is an indication for an improved crystallinity of the BHO films grown and better orientation at higher temperatures. As the substrate temperature is higher than 900 °C, the value of *I*_BHO (002)_/*I*_Hast (220)_ is decreased, which should be caused by the mis-orientation of BHO films. Thus, the optimized substrate temperature about 800 °C should be benefit for BHO films epi-growth.

**Fig. 2:**
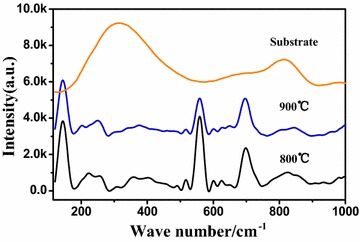
The Raman spectrums of BHO films compared with MgO-buffered substrate

Since the lattice parameters of BaHfO_3_ and MgO are almost similar, it is difficult to distinguish between them in the X-ray diffraction patterns. To confirm the fabrication of BHO and analyse their crystal structure, the Raman scattering experiments are then performed at room temperature, shown in Fig. 2.

According to group theory, an ideal cubic perovskite structure crystal has six selection rules and symmetry species of the fundamental modes, including 3F1u + F2u. The F2u mode is silent and the F1u modes are only infrared active, so there should be no first-order Raman active mode for AETO_3_ crystals in cubic perovskite structure (AE = Ca, Sr and Ba, T = Hf an Zr, space group Pm3m) at room temperature (Karan et al. 2009). Park et al. (1976) found that a distortional noncubic perovskite structures could happen for both CaTO_3_ and SrTO_3_ but not for BaTO_3_ (T = Hf and Zr). In this case, first-order Raman bands are allowed for CaTO_3_ and SrTO_3_. Further research found that this degenerated perovskite structures can also happen in BaZrO_3_ by Ti doping. In this degenerated noncubic structures each of the F1u modes split into two E modes and one A1 mode, and the F2u mode split into one E and two B1 modes. All the A1 and E modes are both Raman and infrared active and the B1 mode is a Raman active (Xie and Zhu 2012).

From Fig. 2, the over damped transverse E1 mode combined with other E1 and A1 Raman modes are obviously detected, located at 145.0, 559.4 and 694.5 cm^−1^, respectively, indicating a degenerated noncubic structures for BaHfO_3_ cap layer films. The sharp A1 modes at 145 and 559.4 cm^−1^ are arisen from the interference of the strong anti-resonance effect, which have been attributed to rotational vibrations associated with the polar BaO_6_ octahedra. Another sharp A1 mode is also detected at 694 cm^−1^, which is attributed to symmetric stretching mode vibrations associated with O atoms. Thus by the observation of the first-order Raman scattering, which is forbidden in a perfect cubic structure, the BHO cap layer films distortions away from the ideal cubic structure are evidenced. This may be caused by the presence of three-dimensional stress from the multi-films system. This stress induced structure degeneration is also occurred in CeO_2_ buffered on Ni–5 at.% W substrate (Lu et al. 2015).

**Fig. 3:**
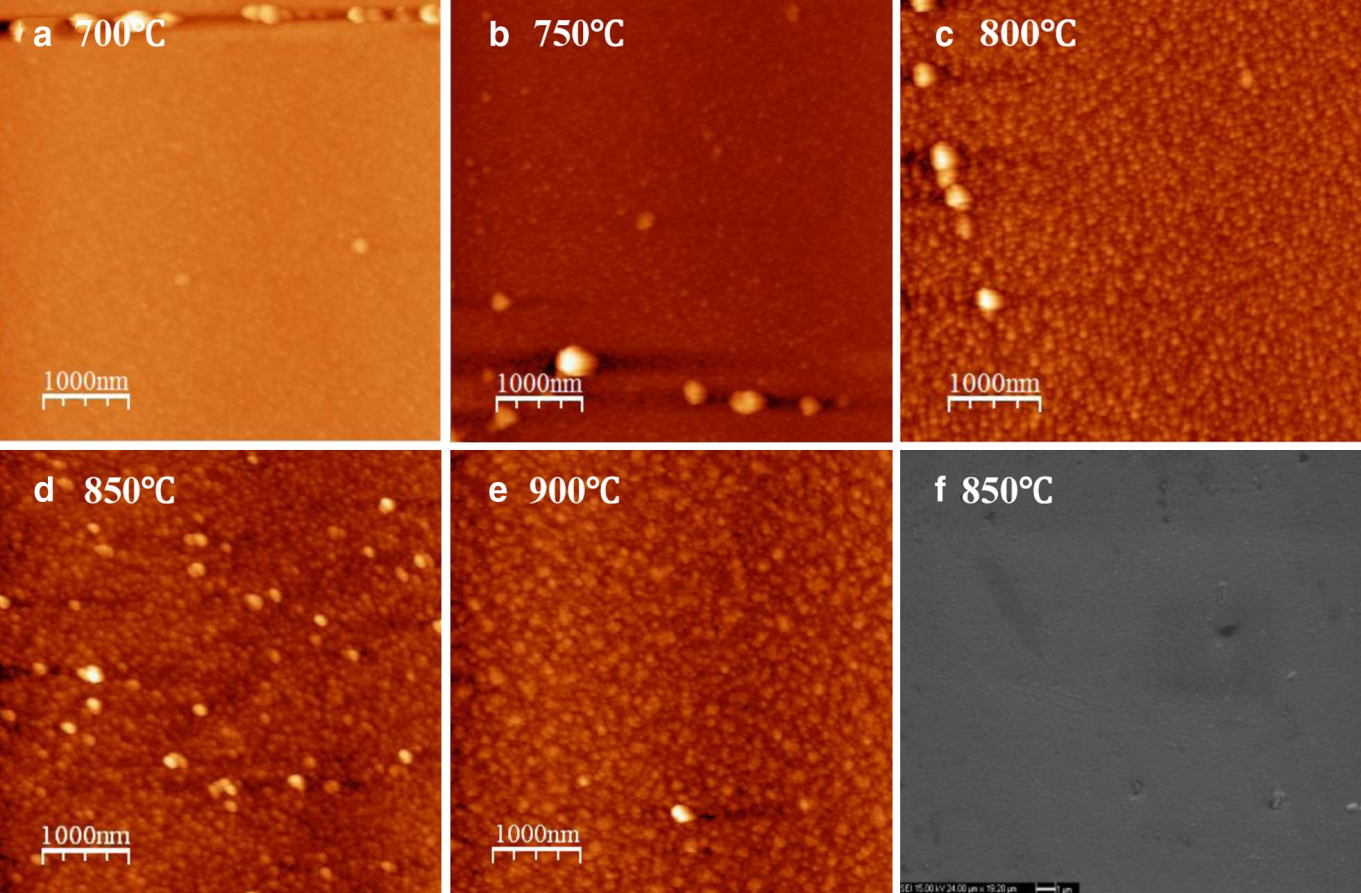
AFM images for the BHO films deposited at **a** 700 °C, **b** 750 °C, **c** 800 °C, **d** 850 °C, **e** 900 °C, and **f** SEM image for the BHO film deposited at 850 °C

Surface morphologies are important for subsequent epi-growth of YBCO films. The surface morphologies of BHO films deposited at different substrate temperature are characterized by AFM and SEM, shown in Fig. 3. From AFM images, smooth, crackfree, pinholefree and compact surface morphologies of BHO films are observed, which should be suitable for subsequently YBCO film epi-growth. The BHO film deposited at 850 °C achieved a relatively smooth surface morphology, with a root mean square surface roughness (RMS) values about 2.7 nm within 5 × 5 μm^2^ area. From SEM image, the morphologies of BHO films are confirmed to be smooth, crack-free and compact. Some particles in line arrange are caused by the crystal boundaries of Hastelloy metal substrate. Energy dispersive X-Ray spectroscopy (EDX) examination shows the atomic ratio of Ba to Hf is about 1.3, confirming the formation of BHO phase, which is corresponded well with the results of XRD pattern and Raman spectrums.

**Fig. 4:**
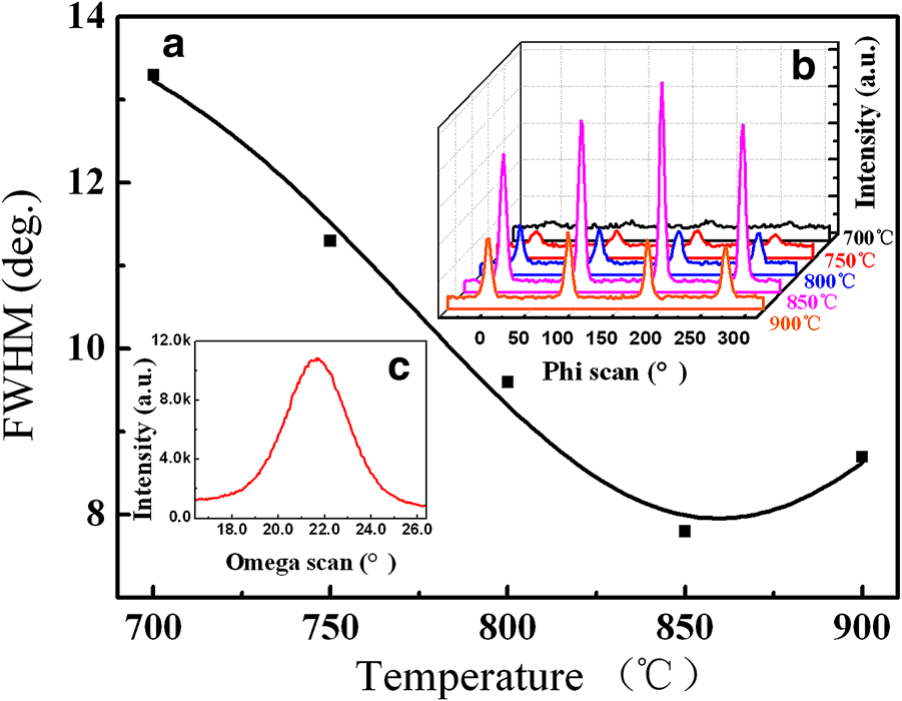
**a** BHO (110) φ-scan deposited at different temperature; **b** the full width at half maximum (FWHM) values of BHO (110) φ-scan deposited at different temperature; **c** BHO (002) ω-scan deposited at 850 °C

The in-plane and out-of-plane texture of BHO films are checked by XRD φ-can and ω–scan, respectively. Figure 4 shows the φ-scan of BHO films deposited at different temperature and the dependence of FWHM values on deposition temperature. The optimized in-plane texture could be achieved at 850 °C, which is fitted well with the results of XRD pattern. The out-of-plane full width at half-maximum (FWHM) values of ω-scan about 3.2° reveal that the BHO films exhibit a strong biaxial texture, indicating BHO a potential cap layer material for YBCO coated conductor.

**Fig. 5:**
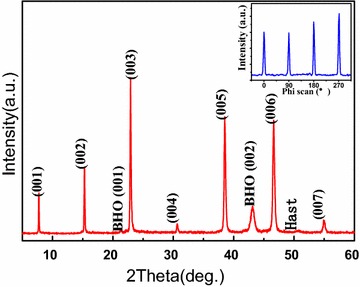
XRD θ–2θ pattern and φ-scan of YBCO films deposited on BHO film

In the next step, typical 300 nm thick YBCO thin films were deposited on the BHO cap layer by PLD to investigate the performance of this coated conductor architecture. The epitaxial relationship of the YBCO layer towards the used BHO template was investigated by XRD θ–2θ pattern and φ-scan measurements, shown in Fig. 5.

**Fig. 6:**
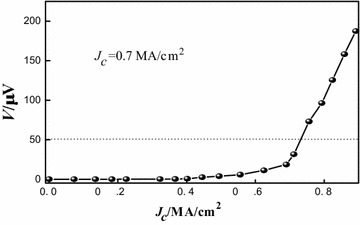
Inductively measured critical current density J_c_ of YBCO film on BHO film

From the θ–2θ XRD pattern of the YBCO film, only YBCO (001) diffraction peak could be detected, showing the YBCO film a well c-axis orientation on such BHO/IBAD-MgO/Y_2_O_3_/Al_2_O_3_/Hastelloy buffer structure. Inset of Fig. 5 shows the (103) φ-scan of the YBCO film. The four symmetric peaks of the phi-scan indicate that the YBCO film is grown cube-on-cube epitaxially relationship to BHO. No additional texture components are observed in the superconducting layer grown on BHO/IBAD-MgO/Y_2_O_3_/Al_2_O_3_ buffered Hastelloy substrate. The in-plane FWHM for YBCO films is about 4.5°, much lower compared with the BHO buffer layers. The critical current density J_c_ of the YBCO coated conductor examined by inductive measurement technique at 77 K in self-field was about 0.7 MA/cm^2^, shown in Fig. 6, demonstrating the BHO a potential cap layer for superconducting coated conductor.

## Conclusion

The BaHfO_3_ films deposited at different substrate temperature were prepared by reel-to-reel magnetron sputtering technique. A stress induced structure degeneration of BHO cap layer films is evidenced by Raman spectrum. The influence of temperature on the texture and surface morphology of BaHfO_3_ films were also investigated. BHO deposited at 850 °C has the relatively best texture. The in-plane and out-of-plane full width at half-maximum values of about 7.8° and 3.2°, respectively, and the BHO film has a dense, smooth, and crack-free morphology. Subsequently epi-growth of YBCO film on BHO cap layer has a pure c-axis orientation with a J_c_ about 0.7 MA/cm^2^ (77 K, self-field), which demonstrates BHO a new buffer layer for coated conductors.
